# Strategies to evaluate the impact of rectal volume on prostate motion during three-dimensional conformal radiotherapy for prostate cancer*

**DOI:** 10.1590/0100-3984.2015.0005

**Published:** 2016

**Authors:** Ana Paula Diniz Fortuna Poli, Rodrigo Souza Dias, Adelmo José Giordani, Helena Regina Comodo Segreto, Roberto Araujo Segreto

**Affiliations:** 1 PhD, Physician Assistant, Unit of Radiotherapy, Centro de Atenção Integrada à Saúde da Mulher - Universidade Estadual de Campinas (CAISM-Unicamp), Campinas, SP, Brazil.; 2 PhD, Physician responsible, Unit of Radiotherapy, Department of Clinical and Experimental Oncology, Escola Paulista de Medicina da Universidade Federal de São Paulo (EPM-Unifesp), São Paulo, SP, Brazil.; 3 PhD, Physicist responsible, Unit of Radiotherapy, Department of Clinical and Experimental Oncology, Escola Paulista de Medicina da Universidade Federal de São Paulo (EPM-Unifesp), São Paulo, SP, Brazil.; 4 Post-doc Fellow, Associate Professor, Unit of Radiotherapy, Department of Clinical and Experimental Oncology, Escola Paulista de Medicina da Universidade Federal de São Paulo (EPM-Unifesp), São Paulo, SP, Brazil.; 5 Private Docent, Associate Professor, Unit of Radiotherapy, Department of Clinical and Experimental Oncology, Escola Paulista de Medicina da Universidade Federal de São Paulo (EPM-Unifesp), São Paulo, SP, Brazil.

**Keywords:** Rectal volume, Prostate cancer, Three-dimensional conformal radiotherapy

## Abstract

**Objective:**

To evaluate the rectal volume influence on prostate motion during three-dimensional conformal radiotherapy (3D-CRT) for prostate cancer.

**Materials and Methods:**

Fifty-one patients with prostate cancer underwent a series of three computed tomography scans including an initial planning scan and two subsequent scans during 3D-CRT. The organs of interest were outlined. The prostate contour was compared with the initial CT images considering the anterior, posterior, superior, inferior and lateral edges of the organ. Variations in the anterior limits and volume of the rectum were assessed and correlated with prostate motion in the anteroposterior direction.

**Results:**

The maximum range of prostate motion was observed in the superoinferior direction, followed by the anteroposterior direction. A significant correlation was observed between prostate motion and rectal volume variation (*p* = 0.037). A baseline rectal volume superior to 70 cm^3^ had a significant influence on the prostate motion in the anteroposterior direction (*p* = 0.045).

**Conclusion:**

The present study showed a significant interfraction motion of the prostate during 3D-CRT with greatest variations in the superoinferior and anteroposterior directions, and that a large rectal volume influences the prostate motion with a cutoff value of 70 cm^3^. Therefore, the treatment of patients with a rectal volume > 70 cm^3^ should be re-planned with appropriate rectal preparation.

## INTRODUCTION

Prostate cancer is the most common visceral malignancy in men^(1)^. Radiotherapy (RT) is one of the main therapeutic modalities for patients diagnosed with prostate cancer, yielding favorable outcomes in terms of local control and overall survival. Computed tomography (CT)-based three-dimensional (3D) treatment planning allows for the use of several radiation fields, assessment of radiation dose distribution to organs at risk, and higher accuracy of the dose delivered to the target volume. Furthermore, 3D treatment planning has already shown to improve biochemical control rates^(2)^. For 3D planning, the International Commission on Radiation Units and Measurements reports 50 and 62 recommend that, in order to assure the delivery of the prescribed dose, the planning target volume (PTV) should be created around the prostate with appropriate margins^(3,4)^.

A major concern in 3D conformal RT (3D-CRT) is inter- and intrafraction prostate motion, as well as uncertainties due to patient positioning and setup error during treatment^(5-7)^. The adequate definition of margins is important for appropriate patient treatment. The magnitude of prostate motion is variable and is mainly related to changes in rectal volume in the anteroposterior direction^(8-10)^.

The purpose of the present study was to evaluate the impact of rectal motion and to correlate rectal volume with prostate motion in anteroposterior direction during 3D-CRT in prostate cancer patients.

## MATERIALS AND METHODS

The present study included 51 patients with biopsyproven adenocarcinoma of the prostate, who had disease limited to the prostate and received a radical course of 3D-CRT in the author's institution. Eligible patients were older than 21 years, with no evidence of metastatic disease, no second malignancy, no history of previous bowel inflammatory disease and not undergoing any immunosuppressive treatment. All the patients were required to acknowledge and sign a term of free and informed consent.

Pretreatment planning was performed with the Acuity Simulator (Varian Medical Systems, Palo Alto, Ca, USA), in the supine position, using a leg holder immobilization device. The isocenter was localized, defined by previous radiography using a 10 X 10 cm field, with the center of the field at the midline of the patient, and bottom limit at the border of the pubic region; and lateral radiography with the anterior limit of the field located posteriorly from 1 to 1.5 cm to the anterior border of the pubic region. Subsequently, a CT scan (5 mm slice thickness) of the pelvis was performed with empty rectum and full bladder. The slices were generated from the iliac crest to the lesser trochanter of the femur and images were sent to the Eclipse planning system at the RT division.

In the treatment planning system, the coxofemoral joints, bladder, rectum, seminal vesicles and prostate were outlined by a single observer. The rectum was delineated from the anal border to the rectum-sigmoid transition, including the whole rectal volume, following the Radiation Therapy Oncology Group (RTOG) recommendations^(11)^. The clinical target volume (CTV) was defined as the prostate and proximal third of the seminal vesicles in patients with < 15% risk of seminal vesicle invasion. For the remaining patients, two CTVs were delineated. In the first plan, the CTV included the prostate and seminal vesicles and, in the second phase, only the prostate. The PTV was established by expanding the CTV by 10 mm in all directions, except posteriorly where it was 8 mm.

Four to six radiation fields were used for treatment planning, the PTV dose ranged from 72 Gy to 73.8 Gy, prescribed in the 95% isodose. For organs at risk, tolerance dose values were followed according to the RTOG protocol^(11)^. Treatment was offered daily, 5 times a week, with daily fractions of 1.8 Gy, using the Varian 600 CD Linac with 6 MV photon energy. The patients were instructed to always come to treatment with a comfortably full bladder and empty rectum.

Between the 10th and 15th and the 25th and 30th fractions, patients were submitted to another pelvic CT scan with a full bladder. Images were transferred to the Eclipse 3D planning system and structures of interest were redrawn and, by means of digital reconstruction radiography, the anterior, posterior, superior, inferior and lateral limits of the prostate were measured in relation to the isocenter. Then, the new measurements were compared with the previous prostate position on the original planning CT scan, and all the variations were recorded.

Additionally, the limits of the anterior wall of the rectum were obtained, to assess a possible correlation between prostate and anteroposterior rectal motion. Variations in rectal volume were also recorded to confirm whether these variations could influence prostate motion and to establish a possible cutoff value for rectal volume on the baseline CT scan.

All data were submitted to descriptive analysis. For quantitative variables, the means and standard deviations were calculated. Pearson's linear correlation coefficients and the Student-t test for independent samples were used to study the association between anteroposterior prostate motion and variations in the anterior wall and volume of the rectum. The receiver operating characteristic curve (ROC) was used to evaluate the correlation between prostate motion and baseline rectal volume at the first CT. A cutoff value for rectal volume at which the greatest influence on prostate motion occurs was established. Statistical analyses were performed with the Statistical Package for the Social Sciences (SPSS) version 17.0 for Windows and the R-Program version 2.11.1. The level of significance was set at p ≤ 0.05.

## RESULTS

The mean age of the patients was 66 years, ranging from 47 to 78 years; 19.6% of the patients were classified as low-risk, 33.3% as intermediate risk, and 47.1% as high-risk disease^(13)^. Variations in prostate motion in all directions are described on Table 1.

**Table 1:** Measurements of prostate variations

Prostate	Mean (mm)	Standard deviation (mm)
Anterior	3.9	3.4
Posterior	4.0	3.2
Superior	5.2	4.6
Inferior	4.2	3.1
Right	2.2	1.8
Left	2.4	2.7
Anteroposterior	3.9	3.3
Superoinferior	4.7	4.0
Left-right	2.3	2.3

Table 2 shows data regarding variations in the anterior wall and volume of the rectum, analyzing the images of three CT scans performed. Such variations in the rectum correlated with anteroposterior prostate motion. As regards influence of the rectal volume on prostate motion, a significant influence was observed in the posterior direction (Table 3).

**Table 2:** Variations in the rectum

Rectum	Mean	Standard deviation
Anterior wall	8.5 mm	8.4 mm
Volume	28.6 cm^3^	35.7 cm^3^

**Table 3:** Influence of rectal variations on prostate motion

Rectum	Prostate	p-value*
Anterior wall	Anterior: 0.139	0.174
	Posterior: –0.019	0.853
Volume	Anterior: 0.074	0.469
	Posterior: 0.211	0.037

* Pearson’s correlation

The cutoff value for rectal volume in relation to anteroposterior prostate motion at the baseline CT was estabilished as < 70 cm^3^ (*p* = 0.045). Patients with rectal volumes ≤ 70 cm^3^ presented a significant prostate motion in the posterior direction (*p* = 0.045) (Table 4).

**Table 4:** Influence of rectal volume on prostate motion

Prostate			
Rectal volume	Posterior variation	Anteroposterior variation	*p*-value *
(cm^3^)	(mean)	(mean)	
≤ 70	0.368	0.34	0.045
> 70	0.421	0.433	

## DISCUSSION

Prostate motion occurred in all directions, with a slightly higher value in the superoinferior direction followed by the anteroposterior direction. Greater motion in the anteroposterior direction, closely followed by the superoinferior direction is reported in the literature. Beard et al. have observed that prostate motion occurs during treatment, and that it may be influenced by the rectal volume. Maximum displacement of the prostate was 13 mm and 8 mm in the posterior and inferior directions, respectively^(13)^. Antolak et al. have assessed prostate motion and obtained margin values of 0.7 cm in the anteroposterior and left-right directions, and 1.1 cm in the superoinferior direction^(14)^. Zelefsky et al. have shown that there is prostate motion in all directions, considerably greater in the anteroposterior and superoinferior directions as compared with the lateral directions, with values of prostate center of mass displacement of 1.2 ± 2.9 mm, 0.5 ± 3.3 mm and 0.6 ± 0.8 mm, respectively^(8)^. Langen et al. have reported the results of a review on prostate motion and concluded that the motion is greater in anteroposterior and superoinferior directions. The standard deviations for anteroposterior motion range from 1.5 to 4.1 mm; for superoinferior motion, from 1.7 to 4.5 mm; and for lateral motion, 0.7 to 1.9 mm^(15)^.

Studies approaching interfraction prostate motion using daily CT imaging in the RT session with the patient immobilized in the treatment position have been published. Frank et al. have demonstrated that the dominant prostate variations occurred in the anteroposterior and superoinferior directions. Such findings were related to the rectal volume change and might influence the CTV dose. The authors emphasize the need for daily directed target localization and/ or immobilization techniques^(16)^. Bylund et al. have shown a mean interfraction prostate motion of 6.7 mm, with the greatest displacement in the anteroposterior direction^(17)^.

Peng et al., using daily CT in 20 patients, reported mean prostate motion of 5.8 ± 3.1 mm for all treatment fractions, with a maximum variation of 20 mm. The authors have also observed the need for replanning in approximately 30% of treatment fractions, as large organ deformation and rotation occurred due to extreme changes in rectal filling^(18)^.

As regards the significant influence of rectal volume variability on prostate motion in the posterior direction, some studies are in agreement with the present study results. Melian et al. have shown that prostate anteroposterior prostate motion is related to variation in rectal volume. Such variation leads to a mean reduction of 6% in the volume of PTV with the 95% isodose^(19)^. Antolak et al. have performed four CT scans with a 2-week interval during RT and found that prostate motion was significantly related to the rectal volume. Furthermore, the rectal volume decreased between the CT-based treatment planning and the first CT during RT^(14)^. The minimum cutoff value of 70 cm^3^ for rectal volume was established on the baseline CT, and values > 70 cm^3^ caused substantial prostate motion in the posterior direction (*p* = 0.045). Data showed similar values as compared with data reported by Zelefsky et al. Those authors have found that patients with a rectal volume > 60 cm^3^ on CT-based treatment planning had significant prostate motion^(8)^. Kupelian et al. have observed that rectal volume > 50 cm^3^ at the planning CT impacts on prostate motion and suggest that the use of daily imaging guidance could eliminate such errors^(20)^. Other investigators have recently reported similar results, showing that the rectal volume impacts on prostate motion and on biochemical management^(21)^.

Finally, data show prostate interfraction motion during 3D-CRT, particularly in the superoinferior and anteroposterior directions. The variability in rectal volume influences prostate motion in the anteroposterior direction, with a cutoff value of 70 cm^3^ for rectal volume at the baseline CT scan. A possible strategy to minimize prostate motion is to repeat the planning CT scan with adequate rectal preparation in patients who have a rectal volume > 70 cm^3^.
